# Supplementary figures and images for: Language models outperform cloze predictability in a cognitive model of reading
Source: PLoS Comput Biol. 2024 Sep 25;20(9):e1012117. doi: 10.1371/journal.pcbi.1012117 (PMC11458034; doi:10.1371/journal.pcbi.1012117)

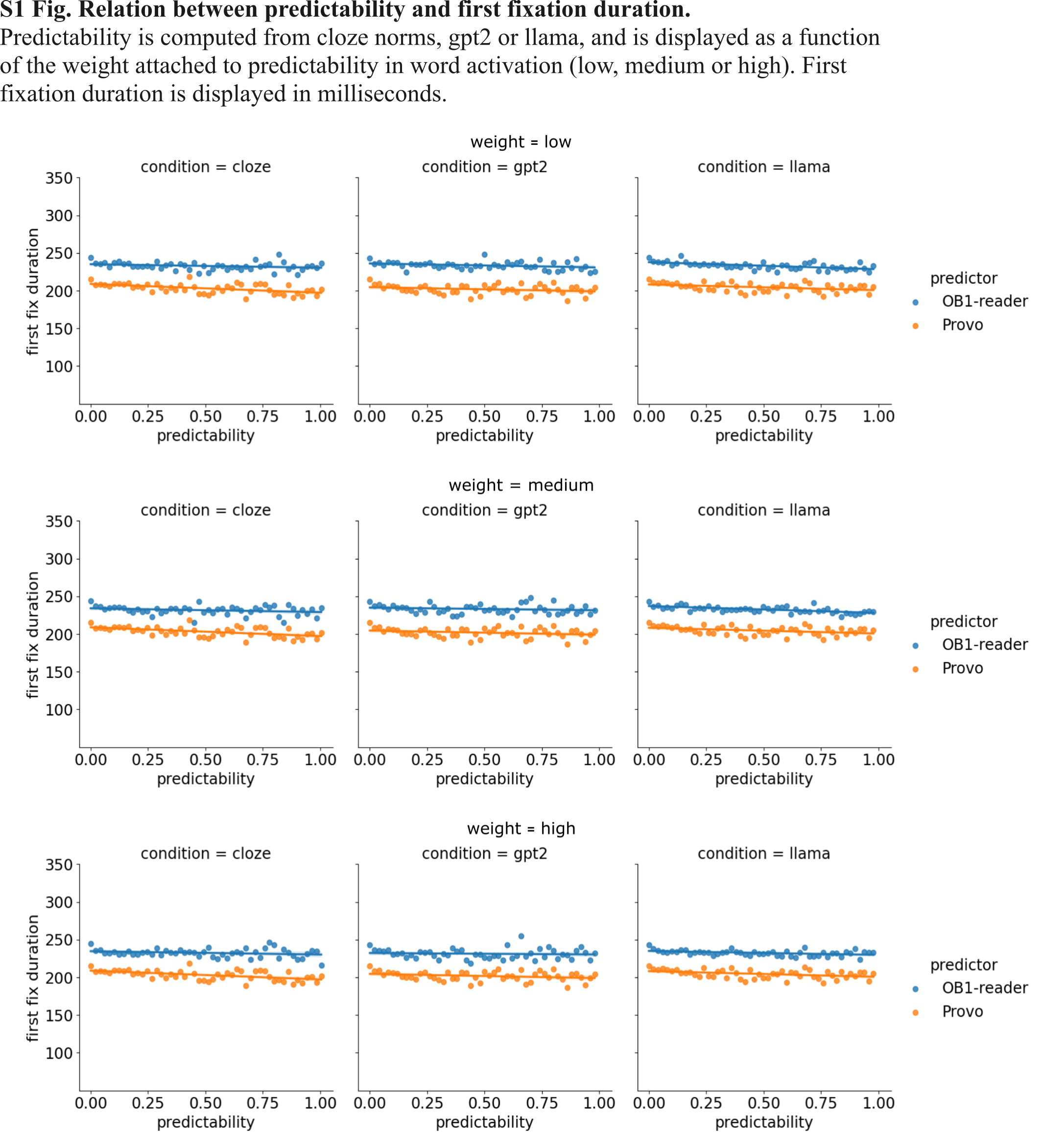

Supplement: S1 Fig — Predictability is computed from cloze norms, gpt2 or llama, and is displayed as a function of the weight attached to predictability in word activation (low, medium or high). First fixation duration is displayed in milliseconds. (TIFF) [file pcbi.1012117.s001.tiff]

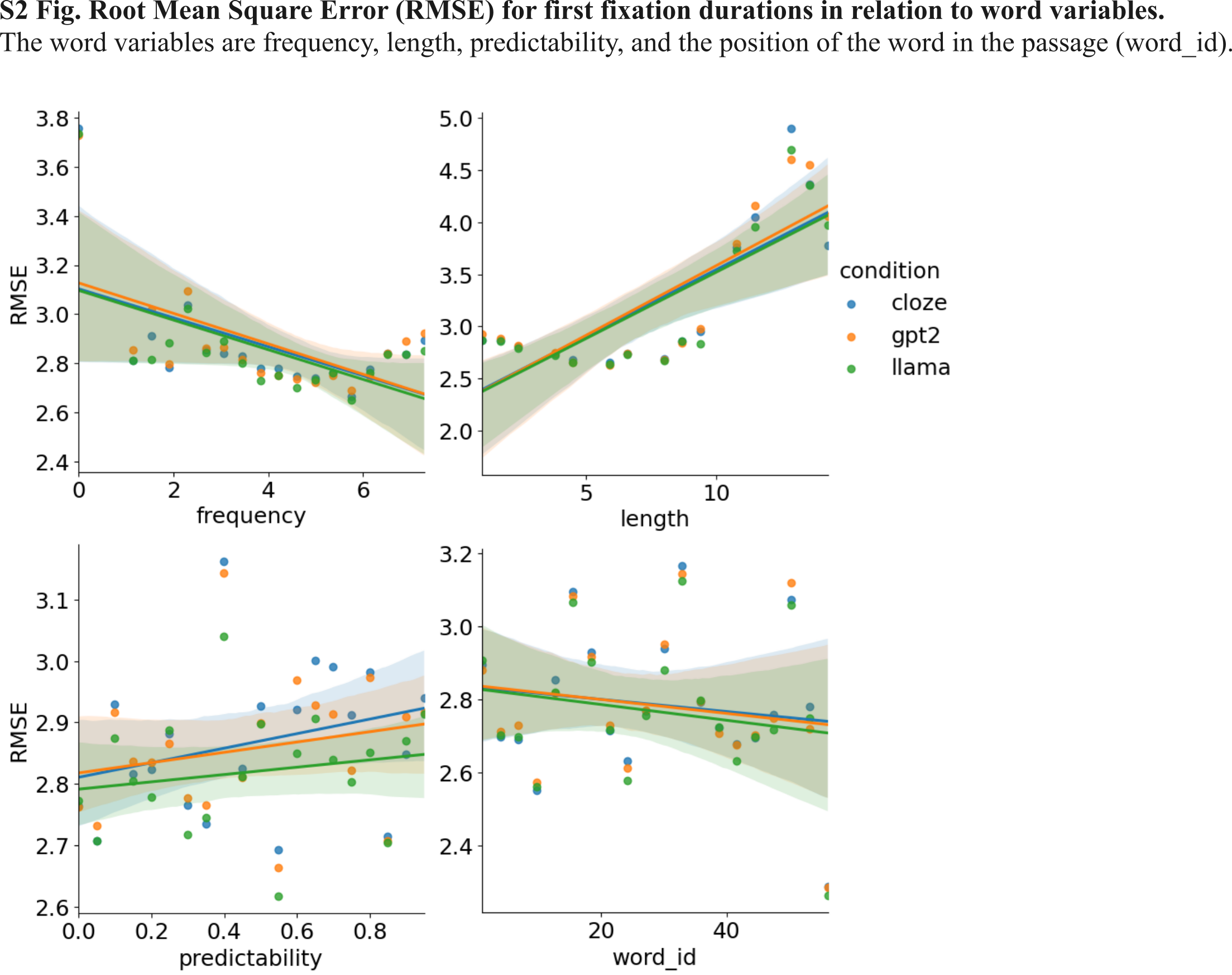

Supplement: S2 Fig — The word variables are frequency, length, predictability, and the position of the word in the passage (word_id). (TIFF) [file pcbi.1012117.s002.tiff]

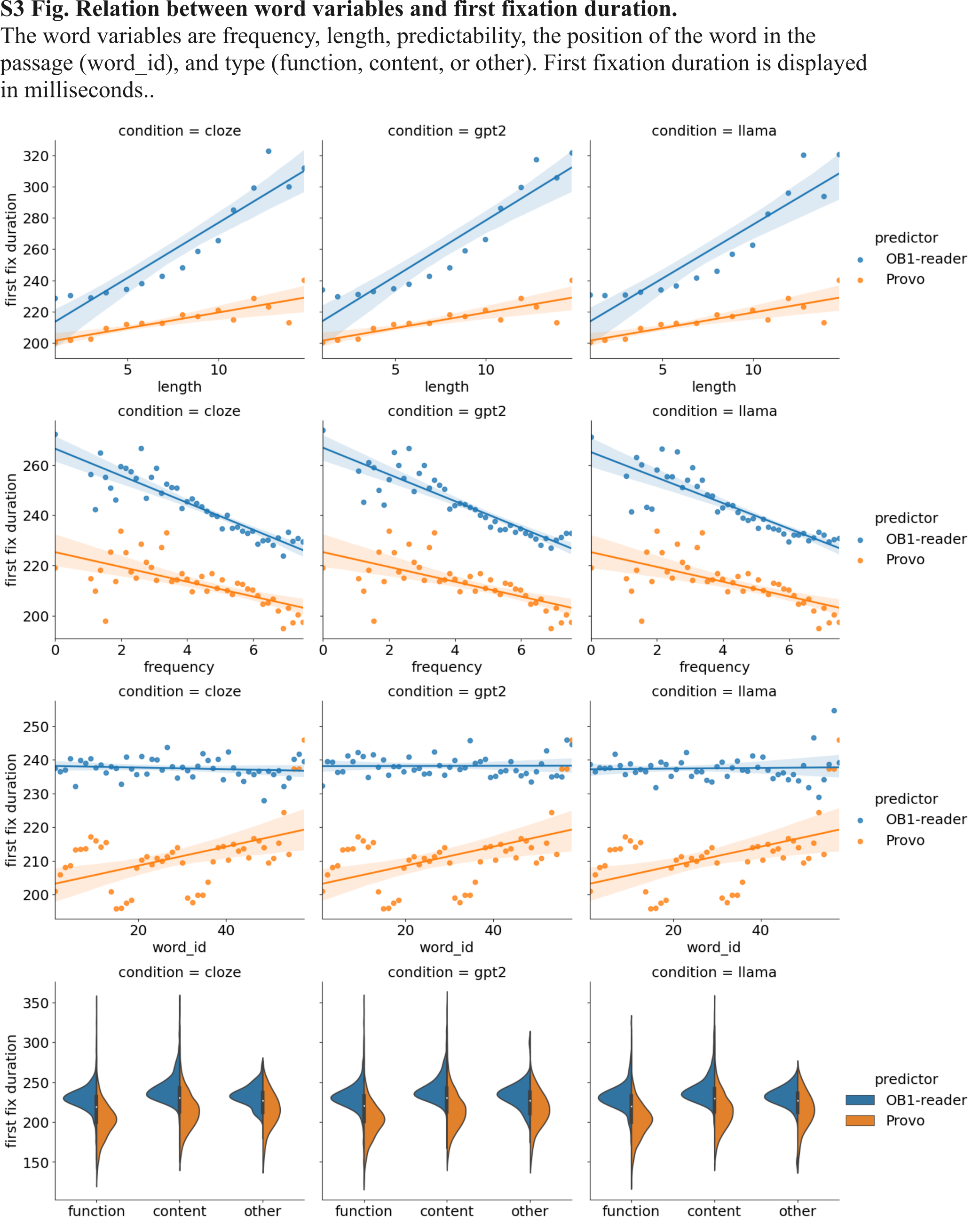

Supplement: S3 Fig — The word variables are frequency, length, predictability, the position of the word in the passage (word_id), and type (function, content, or other). First fixation duration is displayed in milliseconds. (TIFF) [file pcbi.1012117.s003.tiff]

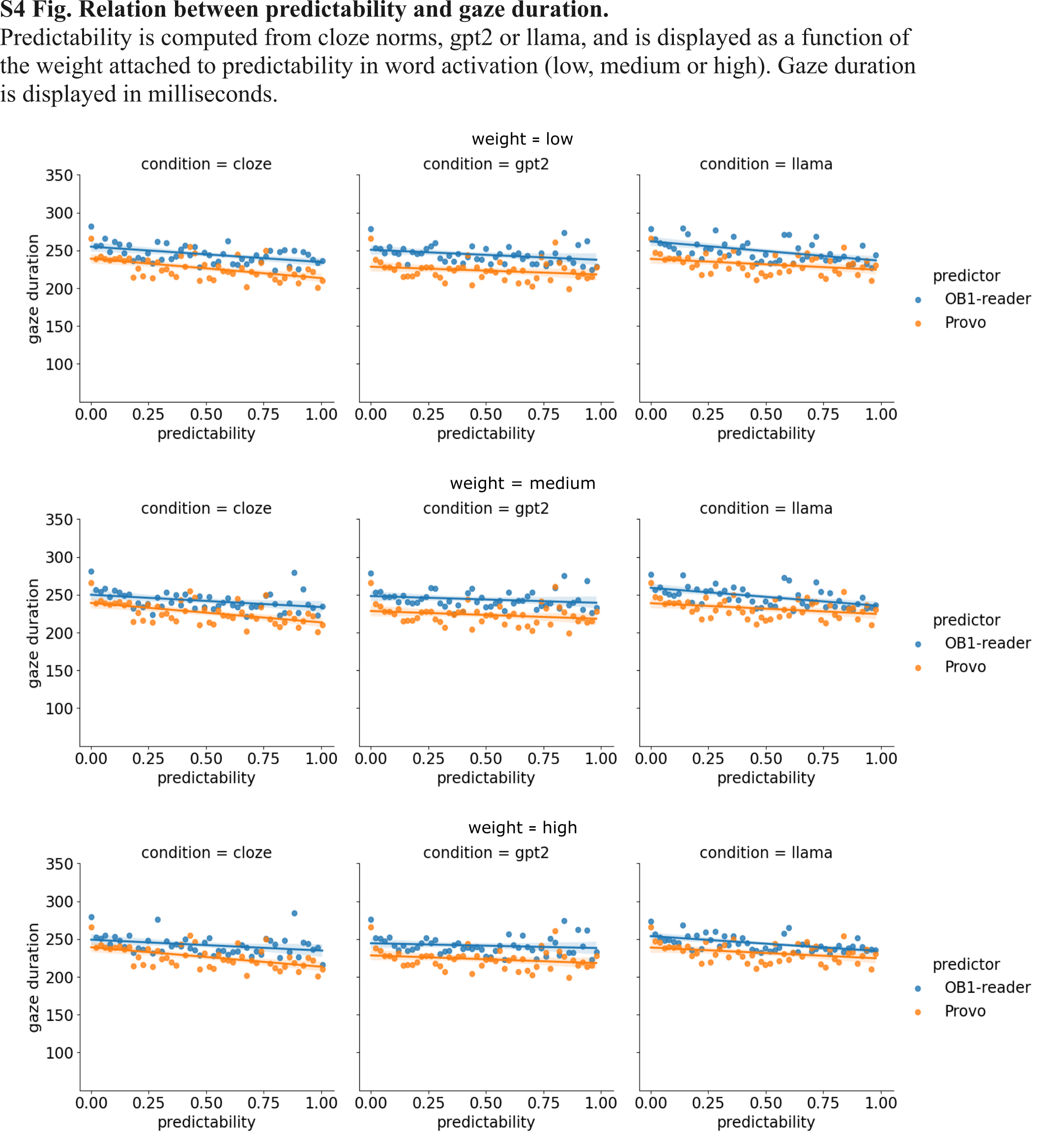

Supplement: S4 Fig — Predictability is computed from cloze norms, gpt2 or llama, and is displayed as a function of the weight attached to predictability in word activation (low, medium or high). Gaze duration is displayed in milliseconds. (TIFF) [file pcbi.1012117.s004.tiff]

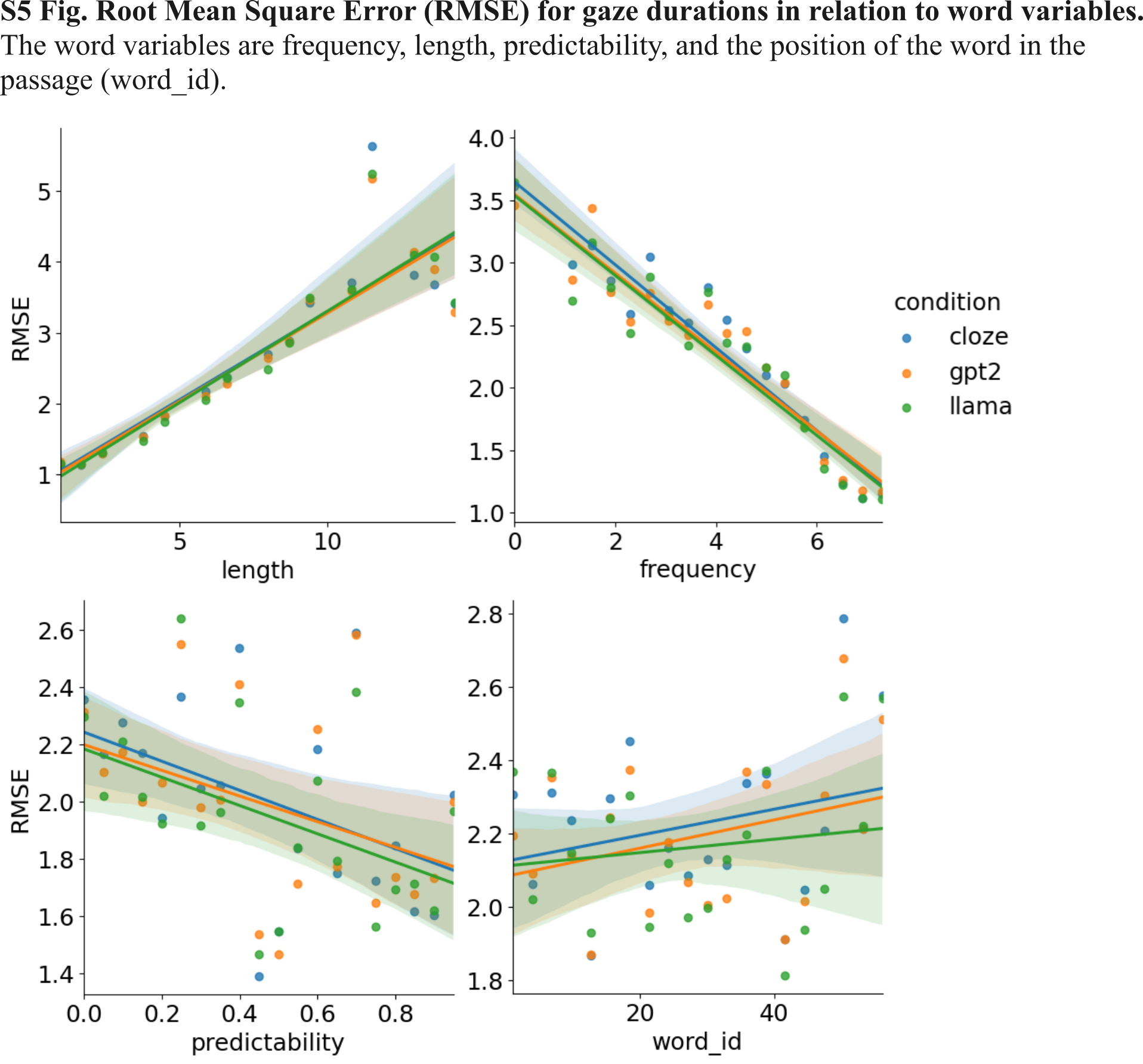

Supplement: S5 Fig — The word variables are frequency, length, predictability, and the position of the word in the passage (word_id). (TIFF) [file pcbi.1012117.s005.tiff]

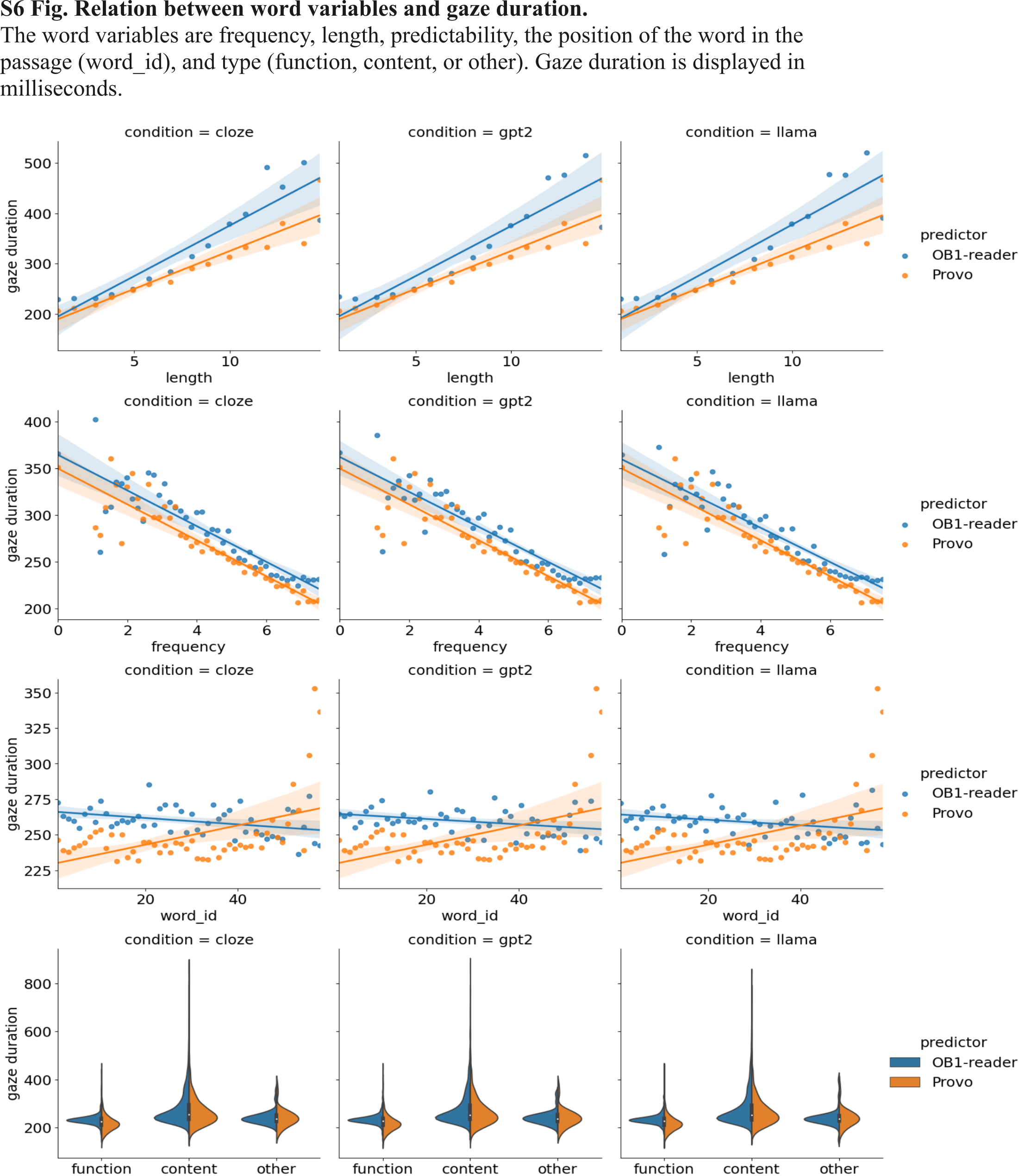

Supplement: S6 Fig — The word variables are frequency, length, predictability, the position of the word in the passage (word_id), and type (function, content, or other). Gaze duration is displayed in milliseconds. (TIFF) [file pcbi.1012117.s006.tiff]

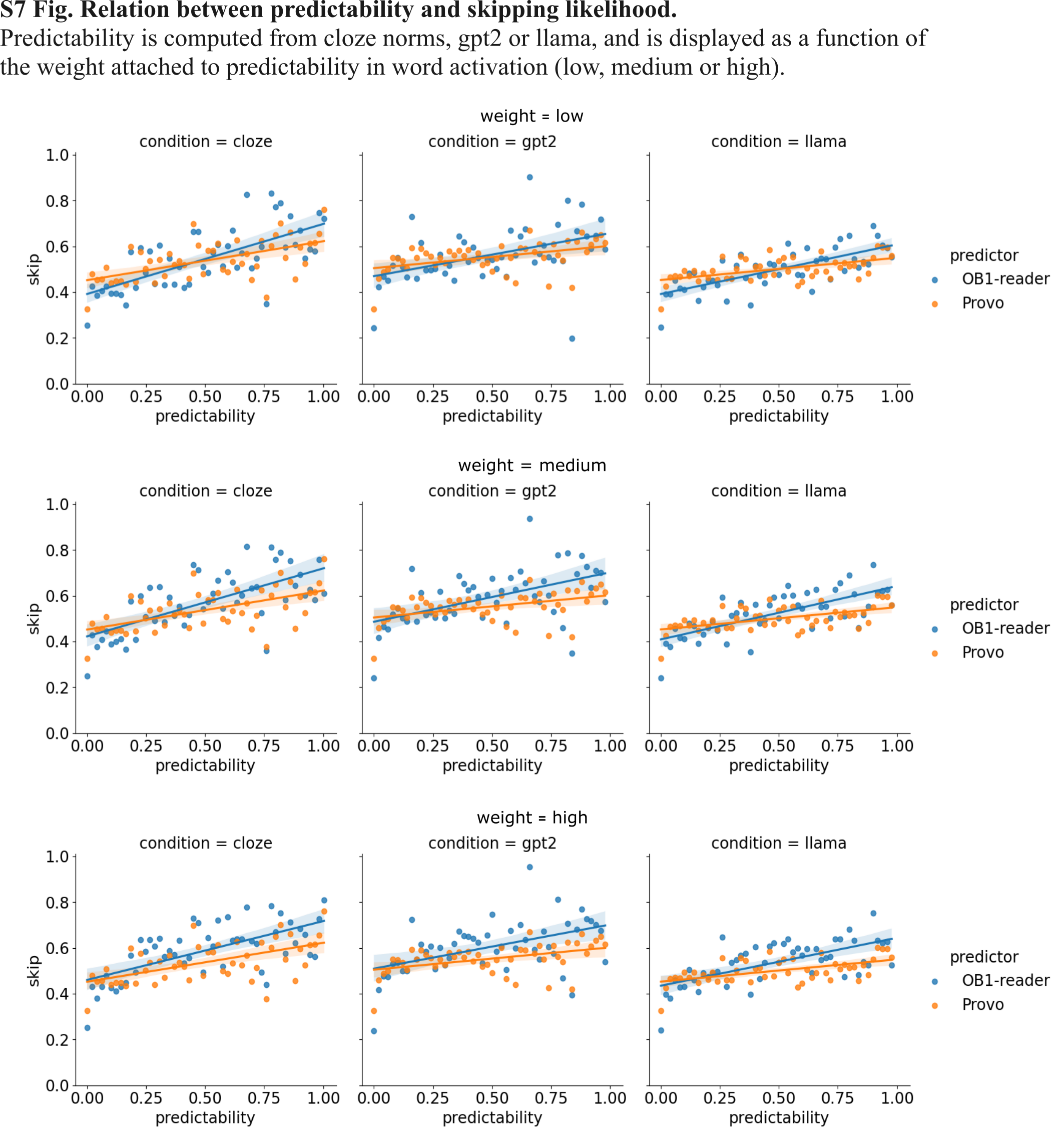

Supplement: S7 Fig — Predictability is computed from cloze norms, gpt2 or llama, and is displayed as a function of the weight attached to predictability in word activation (low, medium or high). (TIFF) [file pcbi.1012117.s007.tiff]

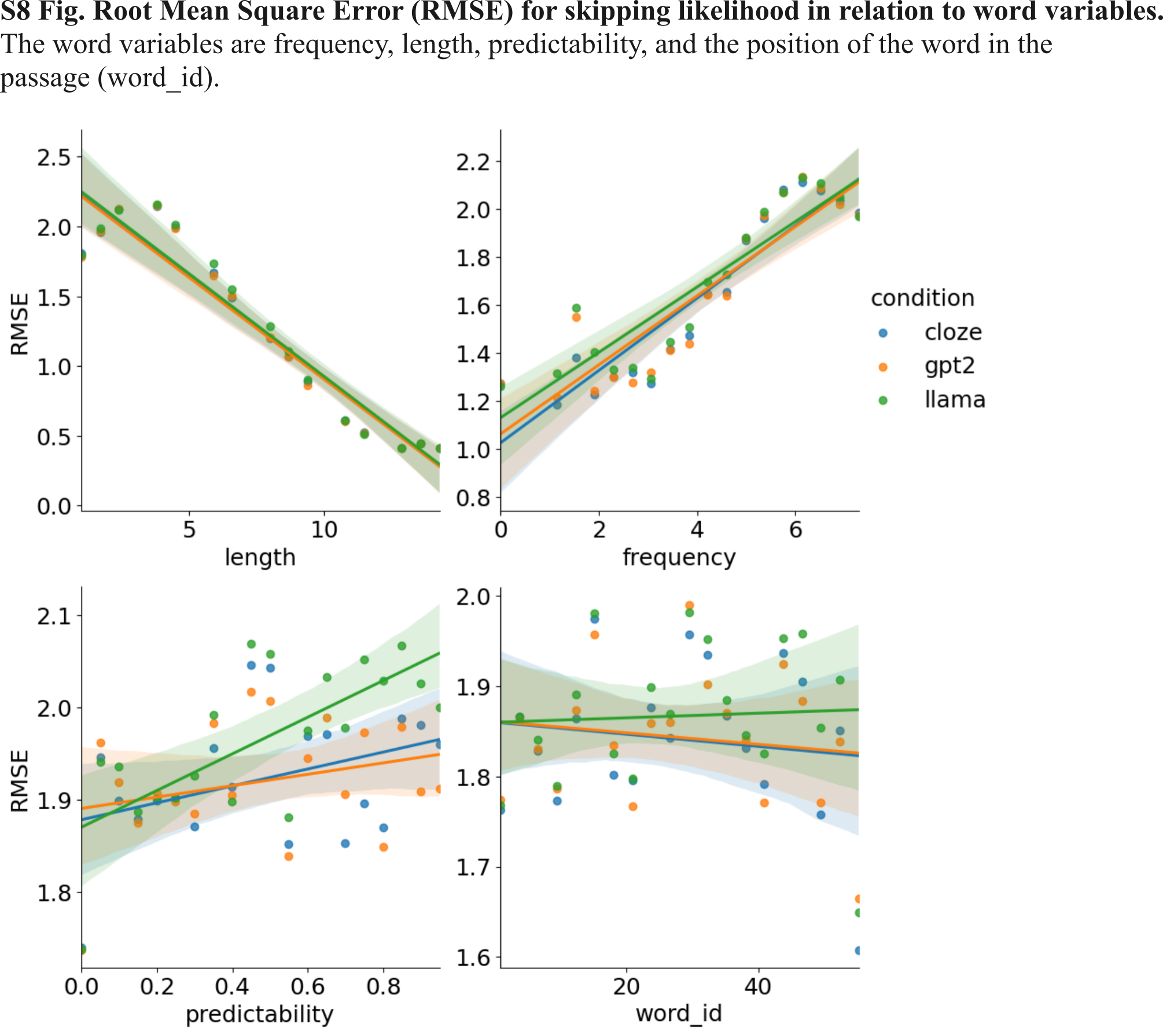

Supplement: S8 Fig — The word variables are frequency, length, predictability, and the position of the word in the passage (word_id). (TIFF) [file pcbi.1012117.s008.tiff]

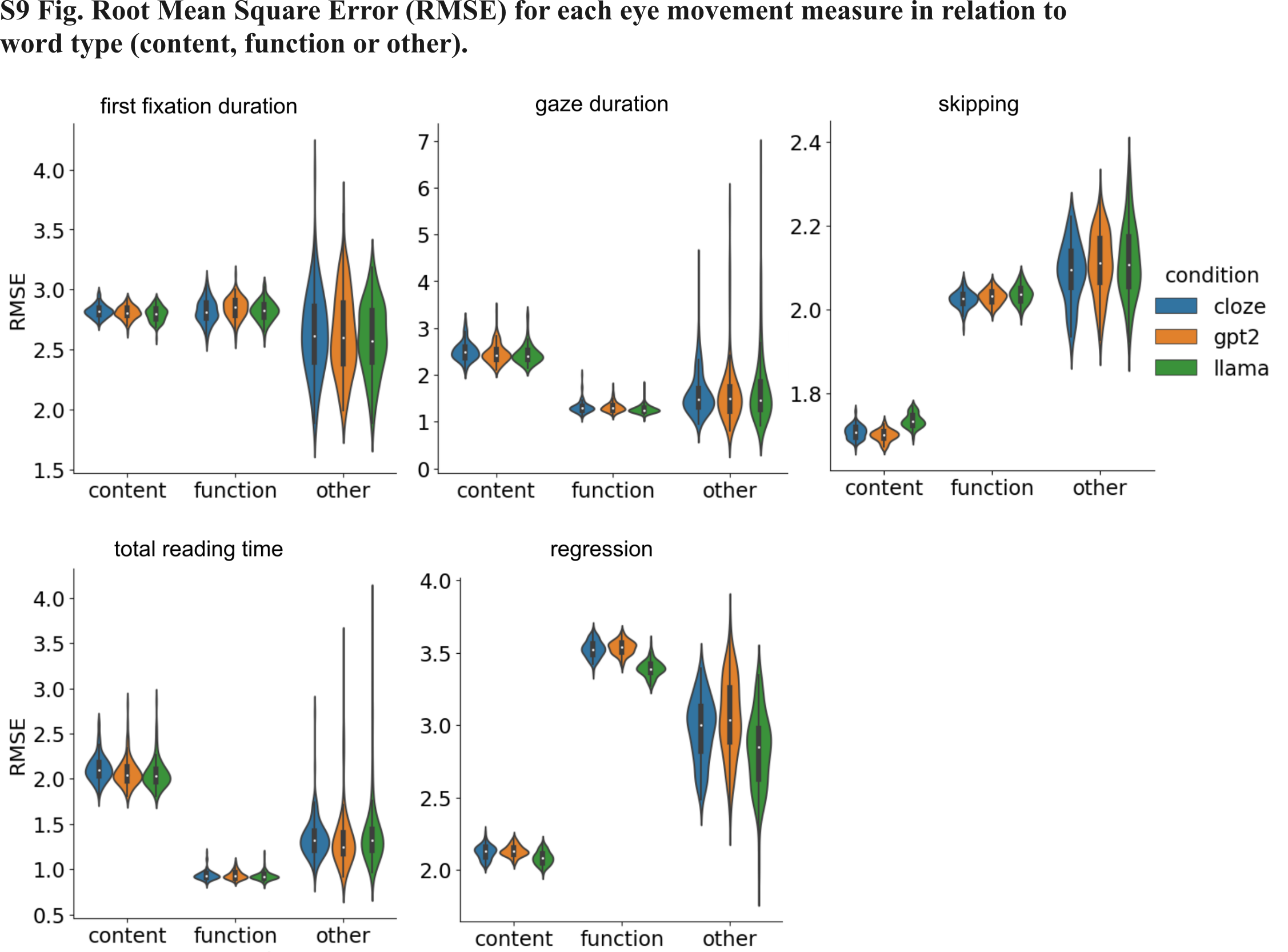

Supplement: S9 Fig — (TIFF) [file pcbi.1012117.s009.tiff]

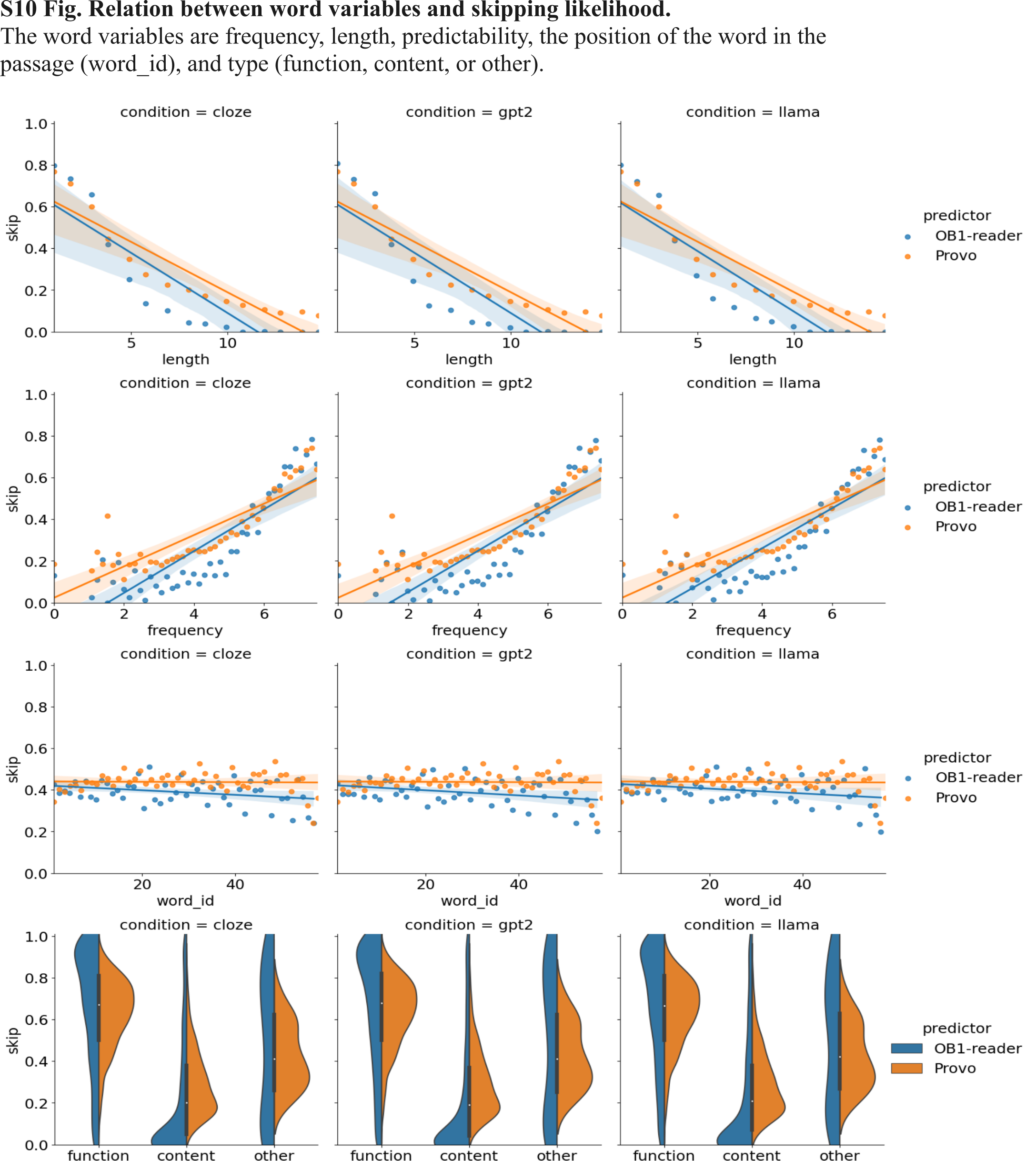

Supplement: S10 Fig — The word variables are frequency, length, predictability, the position of the word in the passage (word_id), and type (function, content, or other). (TIFF) [file pcbi.1012117.s010.tiff]

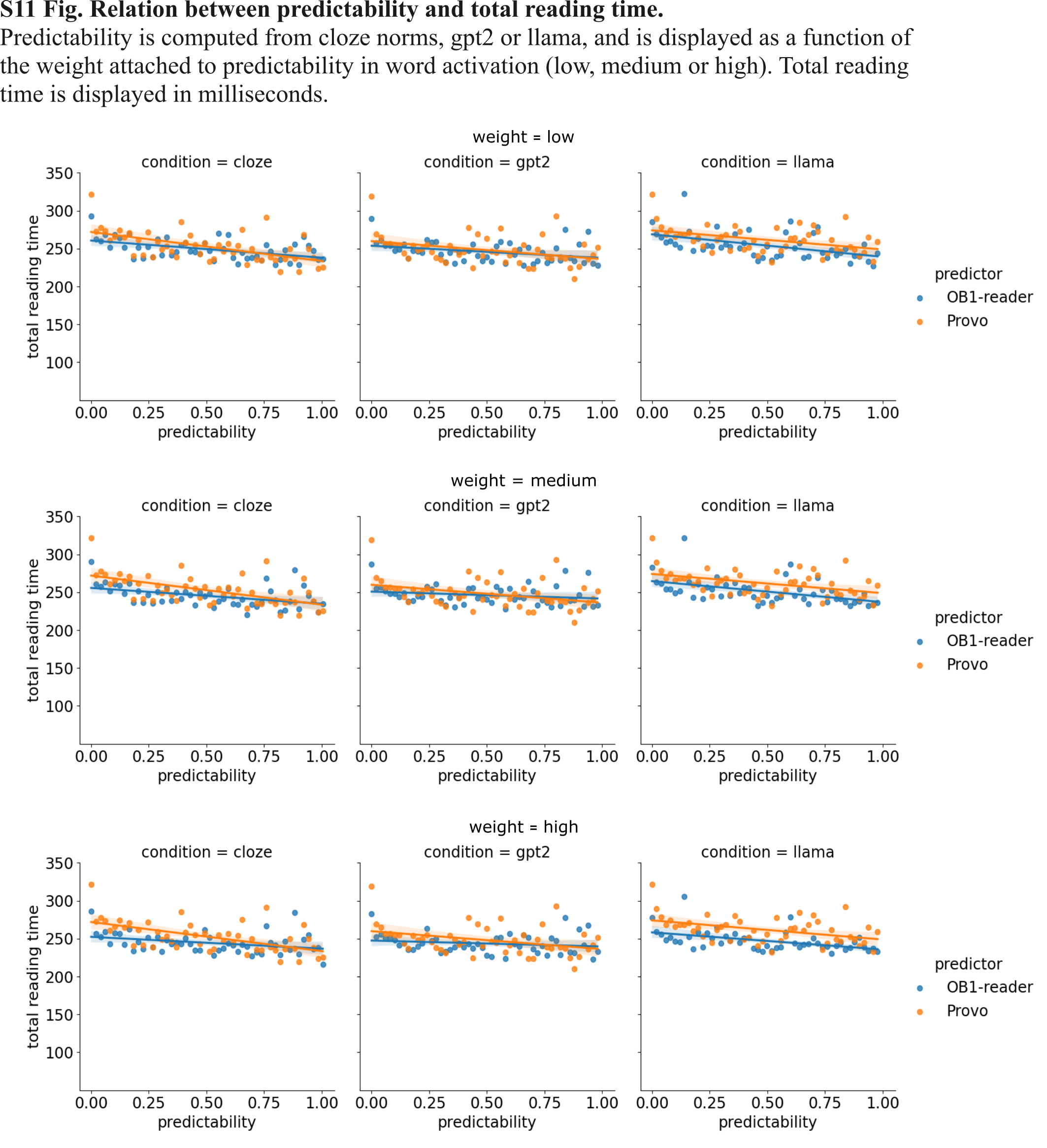

Supplement: S11 Fig — Predictability is computed from cloze norms, gpt2 or llama, and is displayed as a function of the weight attached to predictability in word activation (low, medium or high). Total reading time is displayed in milliseconds. (TIFF) [file pcbi.1012117.s011.tiff]

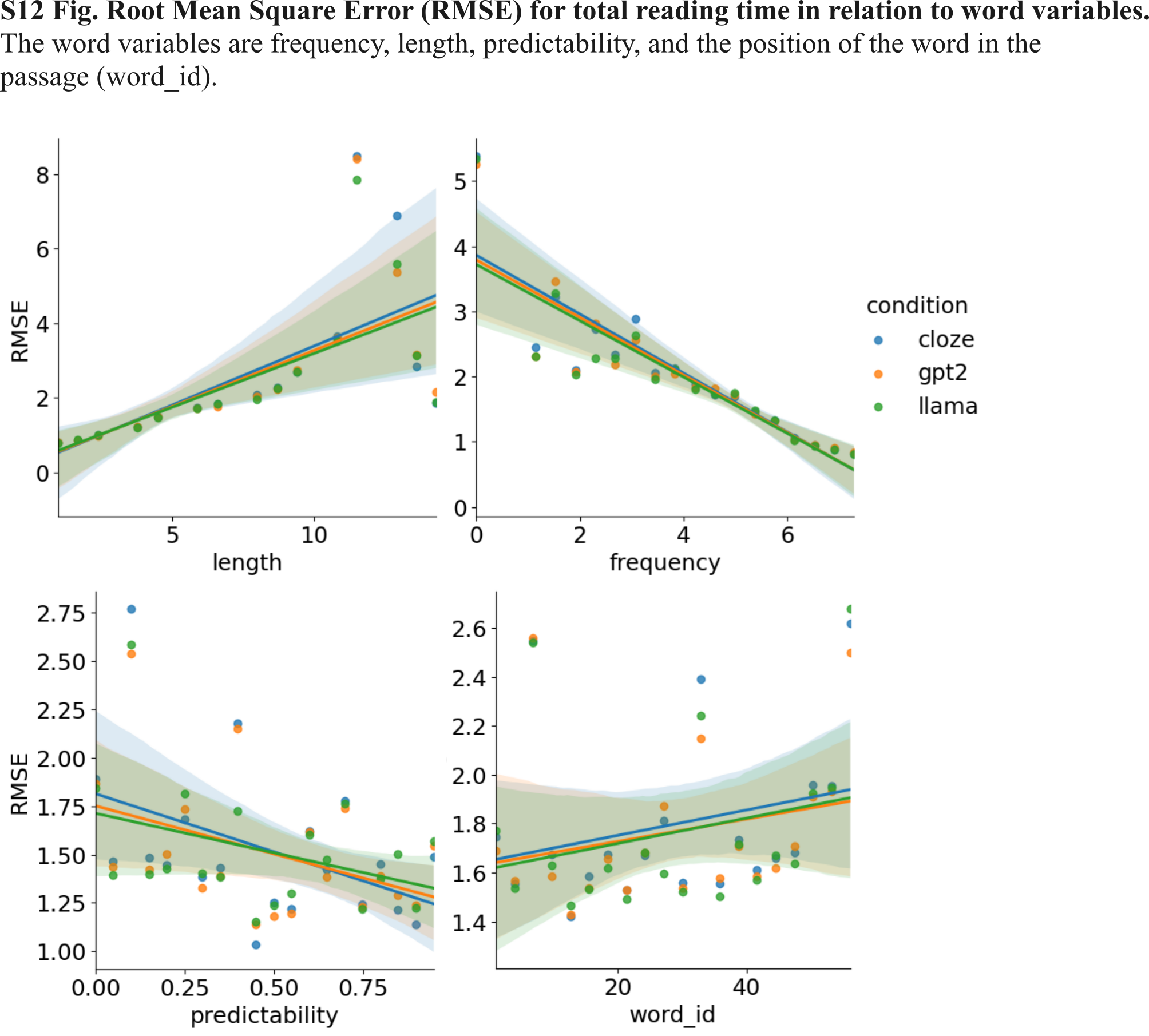

Supplement: S12 Fig — The word variables are frequency, length, predictability, and the position of the word in the passage (word_id). (TIFF) [file pcbi.1012117.s012.tiff]

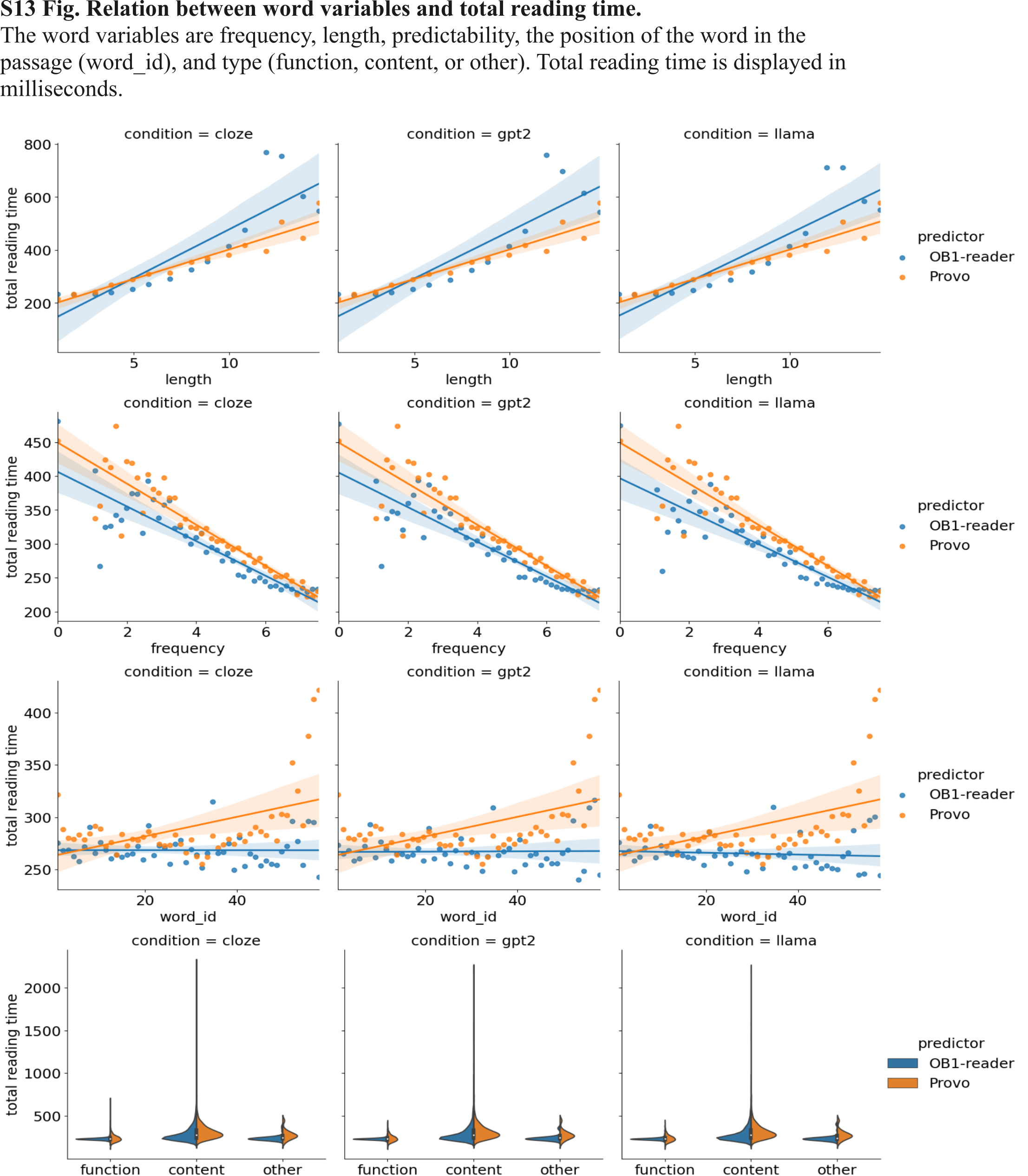

Supplement: S13 Fig — The word variables are frequency, length, predictability, the position of the word in the passage (word_id), and type (function, content, or other). Total reading time is displayed in milliseconds. (TIFF) [file pcbi.1012117.s013.tiff]

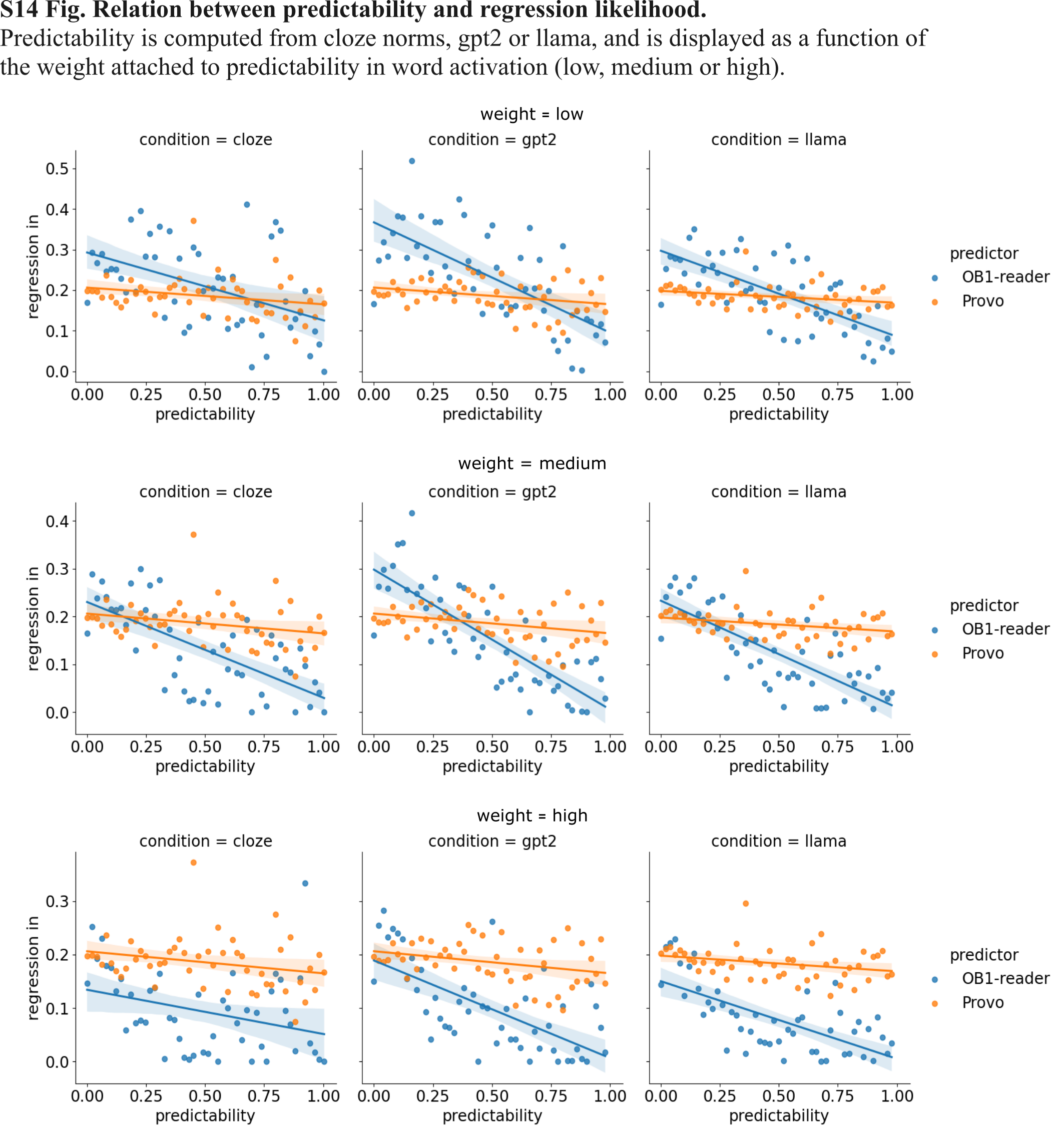

Supplement: S14 Fig — Predictability is computed from cloze norms, gpt2 or llama, and is displayed as a function of the weight attached to predictability in word activation (low, medium or high). (TIFF) [file pcbi.1012117.s014.tiff]

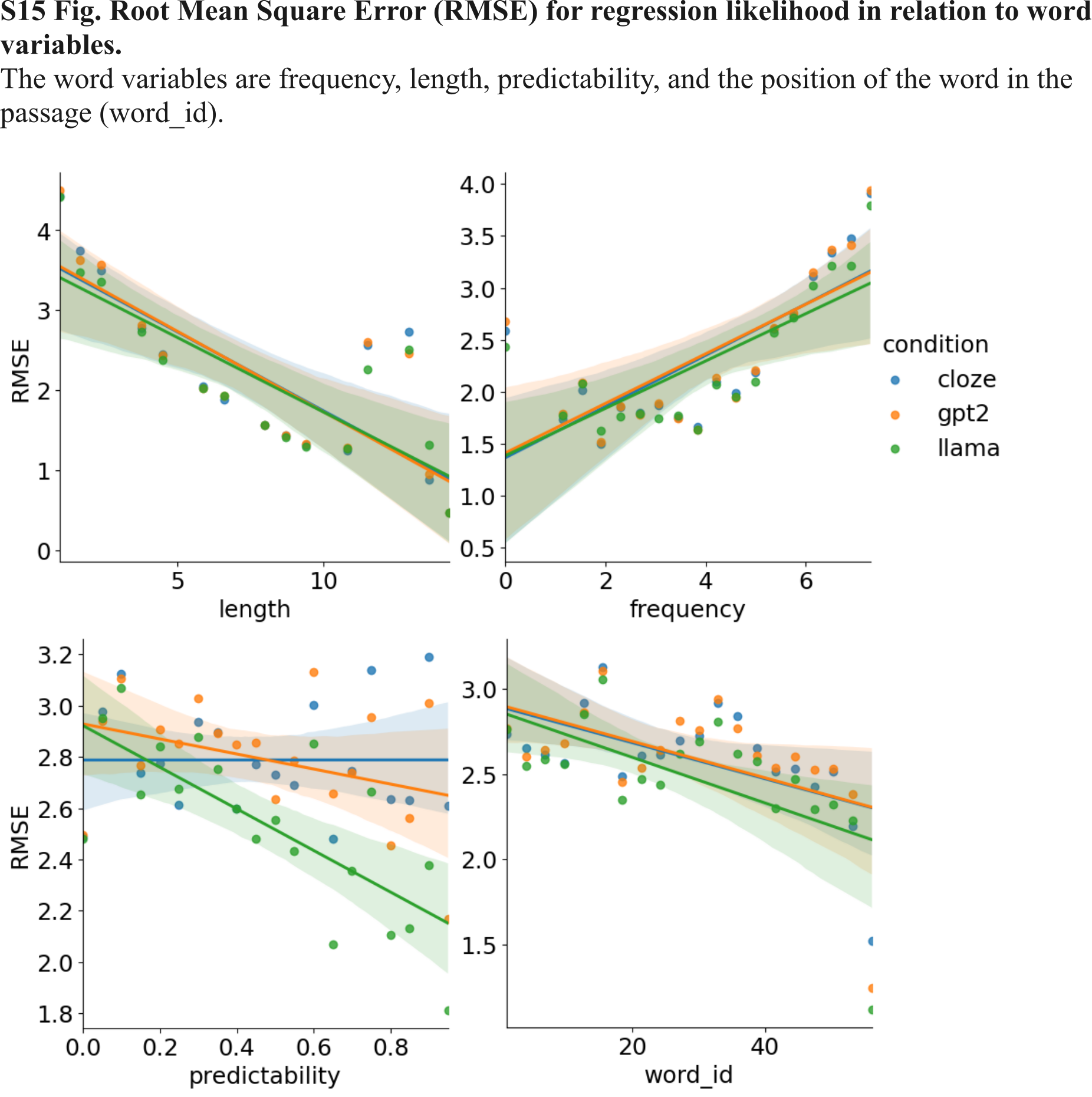

Supplement: S15 Fig — The word variables are frequency, length, predictability, and the position of the word in the passage (word_id). (TIFF) [file pcbi.1012117.s015.tiff]

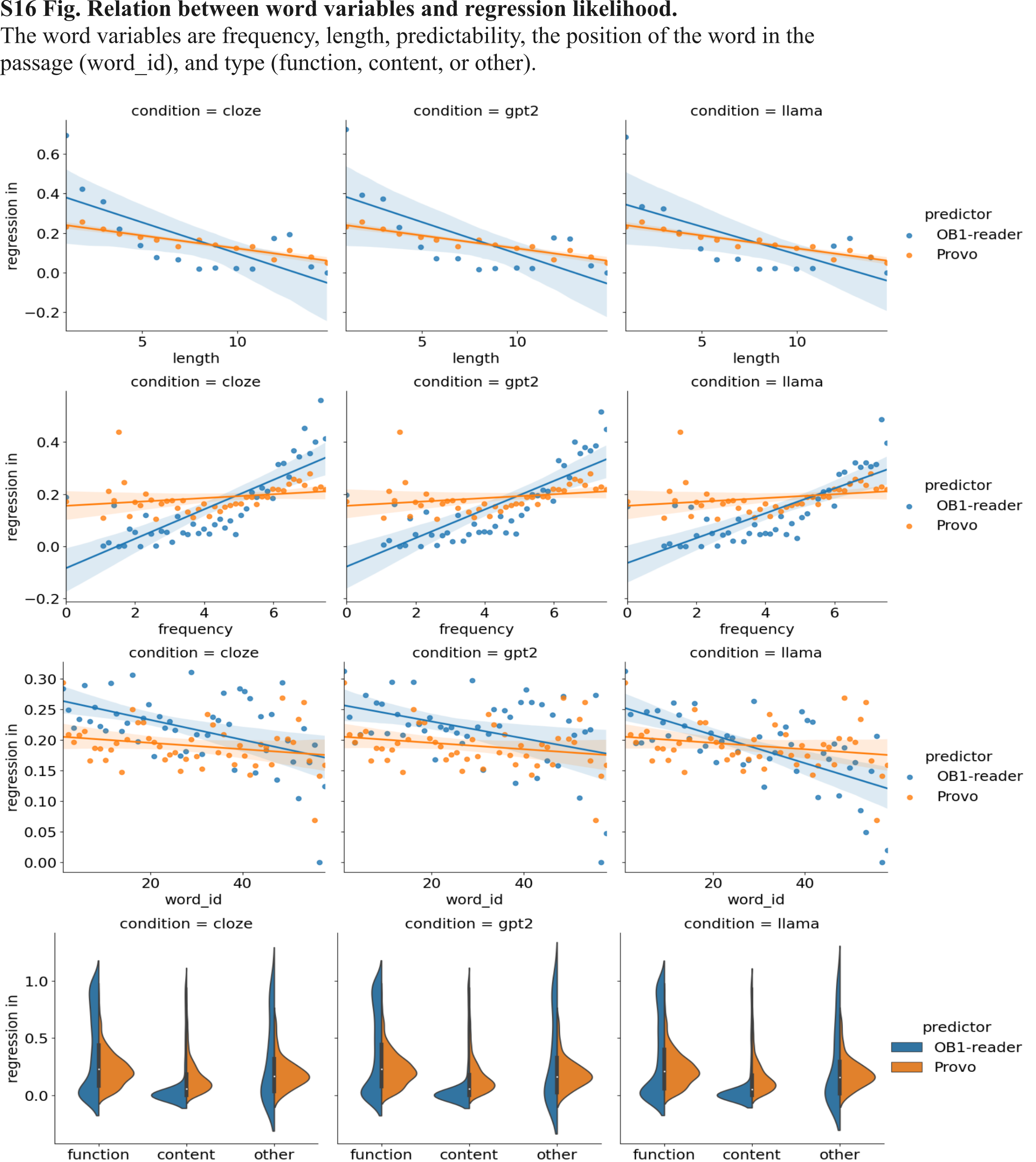

Supplement: S16 Fig — The word variables are frequency, length, predictability, the position of the word in the passage (word_id), and type (function, content, or other). (TIFF) [file pcbi.1012117.s016.tiff]

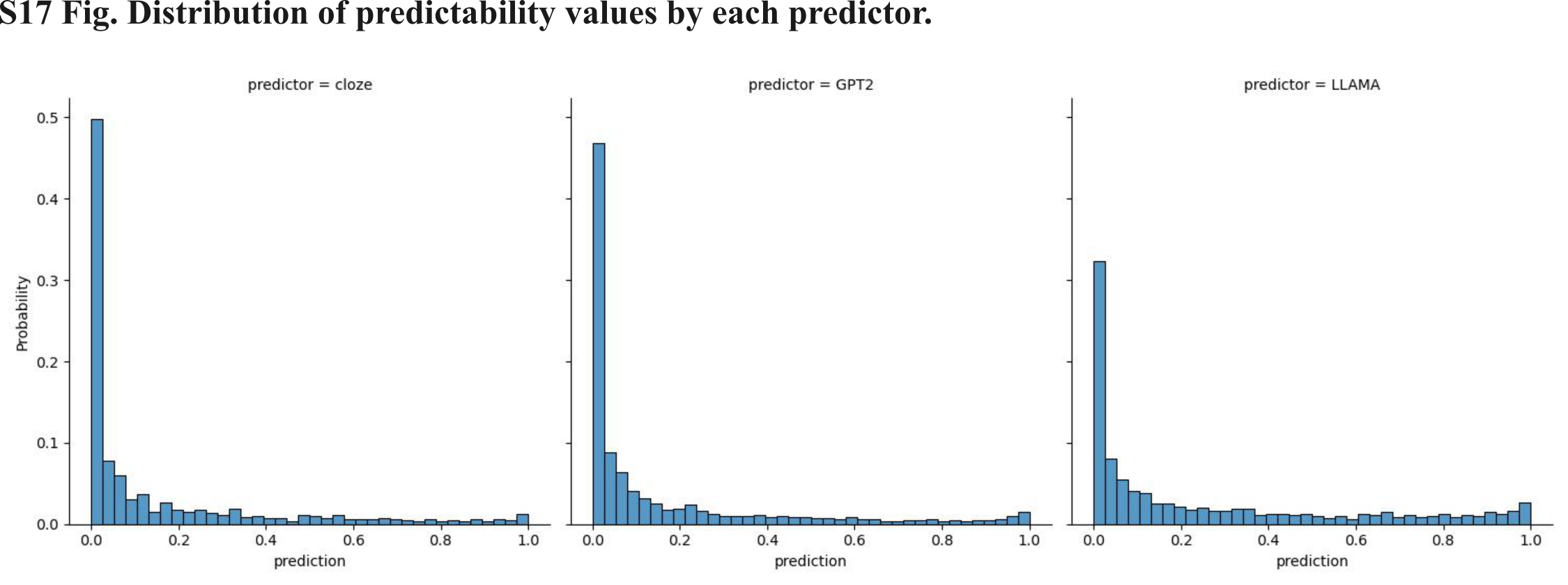

Supplement: S17 Fig — (TIFF) [file pcbi.1012117.s017.tiff]
