# Supplementary material for: Language models outperform cloze predictability in a cognitive model of reading
Source: PLoS Comput Biol. 2024 Sep 25;20(9):e1012117. doi: 10.1371/journal.pcbi.1012117 (PMC11458034; doi:10.1371/journal.pcbi.1012117)
Supplement: S1 Appendix — (DOCX) [file pcbi.1012117.s018.docx]

**S1 Appendix. Accuracy and Correlation Analyses**

Prior to all reading simulations, we validated LLM next-word predictability relative to cloze by measuring the word prediction accuracy of each predictor, i.e. LLaMA, GPT-2 and cloze, as well as the Pearson correlation coefficients between predictability values and eye movement values for each predictor.

Given the predictability threshold and token alignment strategies (see Language Models in Methods), LLaMA and GPT-2 respectively yielded 76% and 64% prediction accuracy against 68% from cloze. We considered a prediction accurate when the text word was among the responses, in the case of cloze, and among the predictions above threshold, in the case of an LLM (note that we did not consider whether the text word was the most responded word in the cloze task or was the top prediction of an LLM). This shows that the LLMs can predict words in the text with a good level of accuracy, comparable to cloze. Although this is not surprising given that LLMs are trained on next-word prediction using many parameters and a massive training data, it suggests that text statistics combined with powerful computation can inform lexical prediction well, relative to humans in a cloze task.

S1 Table shows the Pearson correlation between predictability values and eye movement measures. All comparisons yielded significant correlations (p-value $<= .05$) and in the expected directions. Although predictability and LLM predictability highly correlate with each other ($r=0.57, p<=.0001$ for LLaMA and $r=0.59, p<=.0001$ for GPT-2), predictability estimates derived from GPT-2 exhibited slightly higher correlations with all eye movement measures, relative to predictability estimates derived from either cloze or LLaMA, except regression rate. Since word length and frequency can be confounds, we also measured the correlation between word predictability and word length and frequency (see Table 1). GPT-2 is also the model with the highest correlations between word predictability and word length and frequency. Thus, while word predictability from GPT-2 more strongly correlates with the averaged, word-level eye movements, it may not improve the simulation fits as much, if word length and frequency are main contributors to the observed correlation.

**S1 Table. Pearson correlation coefficients between predictability and eye movements, and between predictability and word length and lexical frequency.**

* means p-value <= .05 and *** means p-value <= .0001. SK = skipping; FFD = first fixation duration; GD = gaze duration; TRT = total reading time; RG = regression.

|  | SK | FFD | GD | TRT | RG | Length | Frequency |
| --- | --- | --- | --- | --- | --- | --- | --- |
| cloze | .29*** | -.17*** | -.23*** | -0.26*** | -0.03* | -.25*** | .30*** |
| llama | .27*** | -.18*** | -.21*** | -0.25*** | -.07*** | -.28*** | .32*** |
| gpt2 | .33*** | -.19*** | -.24*** | -.28*** | -.03 | -.23*** | .25*** |
