# Supplementary material for: Language models outperform cloze predictability in a cognitive model of reading
Source: PLoS Comput Biol. 2024 Sep 25;20(9):e1012117. doi: 10.1371/journal.pcbi.1012117 (PMC11458034; doi:10.1371/journal.pcbi.1012117)
Supplement: Attachment — Submitted filename: response_letter_compt_bio.pdf [file pcbi.1012117.s019.pdf]

Dear Dr. van den Berg and Dr. Marinazzo,

Thank you for the opportunity to improve the manuscript and for the valuable feedback. Hereby we submit the revised work, which all authors have reviewed and approved accordingly.

The most substantial change concerns the addition of implementational details to the methods. We have added information on the materials, model description, and simulations, including a table with the model parameters used in the simulations. Another major change is the inclusion of a systematic analysis comparing the hits and failures of the simulation model in relation to the Provo corpus (as suggested by reviewer 1), and of the simulations using LLMs in relation to those using cloze norming (as suggested by the editor). The analysis highlights differences in performance in relation to linguistic variables (word length, word frequency, word predictability, part-of-speech categories, and word position in the passage). We describe the differences in detail in the Results and provide explanations in the Discussion.

All changes are detailed point-by-point below, with the editor and reviewer comments in italic black font and our responses in regular blue font. We hope to have adequately addressed all concerns and believe the changes resulting from incorporating the reviewer suggestions have significantly improved our manuscript.

Kind regards,

Adrielli Lopes Rego

Editor's feedback

One point that was brought up by two of the reviewers – and which I also scribbled down in my own evaluation of your work – is that the methodology should be described in more detail. In addition to the examples of missing details raised by the reviewer, another one is that it was unclear to me what the input to each simulation was; apparently 55 passages from a corpus were used, but how long were those passages, what did they look like, and how were they chosen?

We have enlarged the methods description and will detail additions in response to reviewer comments below. In response to your comment, we made our materials clearer by adding more details on the passages and why we chose them (page 27):

"We use the full cloze completion and reading time data from the Provo corpus (21). This corpus consists of data from 55 passages (2689 words in total) with an average of 50 words (range: 39-62) and 2.5 sentences (range: 1-5) per passage, taken from various genres, such as online news articles, popular science and fiction (see (a) below for an example passage). The Provo corpus had several advantages over other corpora: Sentences are presented as part of a multi-line passage instead of in isolation (37), which is closer to natural, continuous reading. In addition, Provo provides predictability norms for each word in the text, instead of only the final word (38), which is ideal for studies in which every word is examined. Finally, other cloze corpora tend to contain quite many constrained contexts (which are actually rare in natural reading), while this corpus provides more naturalistic cloze probability distributions (21).

(a) There are now rumblings that Apple might soon invade the smart watch space, though the company is maintaining its customary silence. The watch doesn't have a microphone or speaker, but you can use it to control the music on your phone. You can glance at the watch face to view the artist and title of a song."

A second point that was brought up by two reviewers and also appeared in my own notes was that the manuscript would benefit from a discussion of systematic differences between model fit and empirical data. Would it be possible to say something about why the LLM-based measure leads to lower RMSE? On what kinds of inputs does it perform particularly well and are there cases where it does substantially worse? Answers to those kinds of questions (and those raised by the reviewers) could give important insights that go beyond just comparing performances.

We thank you and the reviewers for this excellent point. We now provide an analysis of the model performance in each condition in relation to word-based linguistic variables, which can be found in the final lines of each sub-section of the Results and paragraphs 3, 4, 5, and 6 of the Discussion. To support the analysis, we added the resulting figures to Supporting Information and we mentioned each figure in the manuscript text.

Related to this, a third important point that was brought up is the question of whether the success of the LLM is purely due to its scale (trained on texts from millions of people vs. the 40 participants from which the empirical Cloze norms were derived)?

We know that one of the important factors that make LLMs powerful is the size of the training data: LLMs need millions of texts, whereas each of us receive way less linguistic input throughout our lives. Thus, in terms of the quantity of linguistic input (and perhaps also its nature – only texts vs. speech, gestures, situations, grounded language, etc.), LLMs and humans differ drastically. It is possible that Cloze norms would be closer to language statistics in the world with a larger sample of participants.

However, at least two factors must be taken into consideration: (i) it has been speculated that millions of samples of each context would be needed for cloze norming to achieve the accuracy of LLMs (Shain et al., 2022), which is hardly feasible; (ii) and, as mentioned in our manuscript, we would still need the assumption that cloze norming reflects real-time predictions in language comprehension, which has been challenged (Smith & Levy, 2011; Staub et al., 2015). Related to (i) is the practical advantage of LLMs in providing a good-enough proxy of predictions in language comprehension without the need for running a cloze task with thousands or millions of participants. In our view, this fact by itself strengthens the value of LLMs in reading research. Moreover, not only LLMs have been shown to provide predictability estimates that fit reading data well: other language models with much smaller training data have been successfully employed in the literature, such as ngram models (Smith and Levy, 2013), and recurrent neural networks (Hofmann et al., 2022). Finally, it is well-known that the success of LLMs is not only tied to the size of its training data: the transformer architecture is behind most of the recent revolution seen in natural language processing (we do not argue, however, that we process language in the same way transformers do).

We hope to have clarified this point, and we added this point in light of your question (page 6, paragraph 2, lines 14-19).

Reviewer #1

General comments. I enjoyed reading this manuscript and thought that the basic idea of using LLMs to generate word-predictability values for reading models was both novel and interesting. For those reasons, I think the manuscript is potentially worthy of publication. Before that can happen, however, the manuscript will require a significant amount of revision to address the concerns outlined below.

We thank the reviewer for his comments on our work. We address his valuable feedback below and are confident that the resulting revision has significantly improved our manuscript.

Major Concerns/Questions:

1. Many of the essential details of OB1-Reader and how the simulations were completed were not provided, making it difficult to evaluate the quality of the simulation results. For example, the authors first mention "pre-cycle activation" on p. 7 but only provide some of the details to understand what this is later, on pp. 24-25. Also, although the authors provide a figure of their model, they do not provide any of the implementational details, but instead assume that readers will consult Snell et al. (2018). The action editor may differ with me here, but my view is that this type of paper should be self-contained, and that whatever model details are required to understand the simulations should be provided.

We apologize for the lack of details on the implementation of the simulations. In line with the editor's suggestion and of another reviewer on describing the methods in more detail, we have revised the Methods and added implementational details (pages 32-37), including a table with the model parameters (page 35). To provide a clear structure to the added details, we have split what was previously the subsection "Reading Simulations" into three subsections: (i) "Model Description", with a more detailed description of OB1-reader and the version used in this study; (ii) "Predictability Implementation", where we describe the predictability mechanism in more detail; and (iii) "Reading Simulations", containing information on the input, output and evaluation specific to the simulations of this study.

Regarding the example given about "pre-cycle activation", we have moved the higher-level explanation from Methods to Main (page 9), as we realize this explanation is needed for understanding the assumptions in the implementation of predictability in OB1-reader introduced in the Main section. In addition, we realize that the term is not the most adequate: "pre-cycle" should indicate that the word receives activation resulting from predictions prior to its fixation, but in parallel processors, such as OB1-reader, all words in the parafovea receive activation prior to their fixation. This means that activation prior to fixation is not exclusive to activation resulting from predictions. We now use the term "predictive activation" instead.

In relation to this, one important detail that needs to be expanded upon is related to the issue of serial versus parallel processing of words. For example, on p. 25, the authors indicate that predictability is "parallel, i.e., predictions can be made about multiple text positions simultaneously." This assumption obviously aligns with the architecture of OB1-Reader but is contrary to models that, like E-Z Reader, assume that words are processed one at a time. In fact, in E-Z Reader, a word's predictability can only be used to facilitate its processing if the preceding words have been both identified and integrated into the sentence representation being constructed (see Reichle et al., 2012, p. 159). This point of contrast should be mentioned because the two models, OB1-Reader and E-Z Reader, represent the end points on an important theoretical continuum.

We agree that the parallel-serial contrast should be mentioned, as it is relevant for the implementation of predictability in OB1-reader. We thank the reviewer for this valuable suggestion and have now made this crucial difference explicit between OB1-reader and serial models of reading (Main, 8/2).

2. On pp. 12-14 and in the Supporting Information, the authors discuss the model fits and how they compare to the observed data from the Provo corpus. However, the authors do not discuss the systematic differences between the two. For example, in Figures 1 and 2 of the Supporting Information, the simulated first fixation and gaze durations are consistently longer than the corresponding observed values. Some explanation of these differences seems necessary.

We thank the reviewer for the excellent point. We have added an analysis of the differences between simulated and observed values and our interpretation as to why (please see the final lines of each sub-section under Results; the second, third, fourth and fifth paragraphs of the first sub-section under Discussion; and the figures under Supportive Information mentioned in the added text). The analysis consisted of comparing the model error in fitting human data and comparing raw differences between simulated and human values in relation to word-level linguistic variables (length, frequency, predictability, part-of-speech, and position in the passage). In general, we observed a failure of the model to fit more difficult words: more error was observed for the reading times of longer, less frequent, less predictable, and content words, because it reads them more slowly than humans do, and it also skips and regress to those words less often. Interestingly, the reverse pattern was observed for skipping and regression, where the model generates more error for shorter, more frequent, and function words, with more skipping of function words and more regression to shorter and frequent words relative to humans. When comparing LLMs to cloze, we observed an advantage of LLaMA in fitting gaze duration, total reading time and regression rates of more predictable words. In contrast, we observed a disadvantage of LLaMA in fitting skipping rates of more predictable words. We provide explanations for these differences in the Discussion.

3. I'm not an expert on LLMs, but my understanding is that part of their utility reflects the fact that they've been trained on huge volumes of text, perhaps making their "experience" (using the authors' term; see p. 6) equivalent to that of many readers (as opposed to, e.g., what would be expected of a single reader). In contrast, the empirical cloze norms taken from the Provo corpus were based on an average of only 40 participants (p. 19). If that's the case, would the model's performance using cloze norms *be more equivalent to the model's performance using LMMs if the norms had been based on a much larger sample? Some explanation seems necessary. And if my intuition here is incorrect, then a discussion of precisely why might strengthen the authors' argument for the value of using LMMs.*

Since the editor has made a similar point, we repeat our answer here:

"We know that one of the important factors that make LLMs powerful is the size of the training data: LLMs need millions of texts, whereas each of us receive way less linguistic input throughout our lives. Thus, in terms of the quantity of linguistic input (and perhaps also its nature – only texts vs. speech, gestures, situations, grounded language, etc.), LLMs and humans differ drastically. It is possible that Cloze norms would be closer to language statistics in the world with a larger sample of participants. However, at least two factors must be taken into consideration: (i) it has been speculated that millions of samples of each context would be needed for cloze norming to achieve the accuracy of LLMs (Shain et al.,

2022), which is hardly feasible; (ii) and, as mentioned in our manuscript, we would still need the assumption that cloze norming reflects real-time predictions in language comprehension, which has been challenged (Smith & Levy, 2011; Staub et al., 2015). Related to (i) is the practical advantage of LLMs in providing a good-enough proxy of predictions in language comprehension without the need for running a cloze task with thousands or millions of participants. In our view, this fact by itself strengthens the value of LLMs in reading research. Moreover, not only LLMs have been shown to provide predictability estimates that fit reading data well: other language models with much smaller training data have been successfully employed in the literature, such as ngram models (Smith and Levy, 2013), and recurrent neural networks (Hofmann et al., 2022). Finally, it is well-known that the success of LLMs is not only tied to the size of its training data: the transformer architecture is behind most of the recent revolution seen in natural language processing (we do not argue, however, that we process language in the same way transformers do). We hope to have clarified this point, and we added this point in light of your question (page 6, paragraph 2, lines 14-19)."

Minor Concerns: These suggestions are admittedly nitpicky but intended to be helpful [page/paragraph/line(s)].

We thank the reviewer for the thorough feedback. All the typos mentioned were fixed and all the suggestions for removal were taken. We elaborate on some of the points which required more changing below.

1. General comment: I had the impression that the manuscript was written in haste, with minimal effort to proof it for typos.

We apologize for the typos and highly thank the reviewer for taking the time to proof-read our work in such detail.

2. 2 (abstract)/1/2: Remove "yet".

We removed it accordingly.

3. 3/1/2: "provide" --> "provides"

We corrected it accordingly.

4. 3/1/penultimate sentence: The sentence beginning "In the short term," sound empty and should be expanded or removed.

We agree with the reviewer. We have removed the sentence.

5. 4/1/6: Add two references: (1) Reilly & Radach (2006); and (2) Li & Pollatsek (2020).

We have updated it accordingly.

6. 4/1/7 (and elsewhere throughout manuscript): "Cloze" is typically not capitalized.

Indeed. We have updated it throughout the manuscript.

7. 4/3/4 (and elsewhere): Use the Oxford comma with lists of three or more items to avoid ambiguity.

Thank you for the suggestion. We revised the manuscript accordingly, for instance:

"These attempt to explain how the brain guides the eyes, by combining perceptual, oculomotor, and linguistic processes."

8. 5/1/2: "EZ-reader" --> "E-Z Reader"

We apologize for the typo. We fixed it throughout the manuscript.

9. pr: Although what is said here is technically true, in E-Z Reader, the cloze values are "fixed" only because the model doesn't provide a deep account of language processing. As such, I don't conceptualize cloze values as being static (as suggested by the authors) but instead view this assumption as a proxy. Some acknowledgment of this seems necessary. (And not just for my model, but for the others, too.)

We agree with the reviewer about the rationale behind using "fixed" or "static" predictability estimates. We have revised this paragraph and added this consideration (5/1/final sentences):

"These models ignore the predictability of words that do not appear in the stimulus text, even though they may have been predicted at a given text position and assume a one-to-one match between the input and the actual text for computing predictability values. Because the models do not provide a deeper account of language processing at the syntactic and semantic levels, they cannot allow predictability to vary dynamically as text processing unfolds. Instead, predictability is computed prior to the simulation and fixed."

10. 5/2/6-7: Move the parenthetical example to the end, as a new sentence.

We updated it accordingly.

11. 6/1/7: "is determined" --> "are determined"

We fixed it accordingly.

12. 6/1/10: "the phrase "as well as (17), or outperform (18)" should be re-written and expanded, with more details provided (i.e., not just the citations).

We have edited it and provided more detailed descriptions on the studies mentioned (7/2):

“Importantly, language models have recently been shown to perform as well as, or even outperform, predictability estimates derived from cloze tasks in fitting reading data. Shain and colleagues (20) found robust word predictability effects across six corpora of eye-movements using surprisal estimates from various language models, with GPT-2 providing the best fit. The effect was qualitatively similar when using cloze estimates in the corpus for which they were available. Another compelling bit of evidence comes from Hofmann and colleagues (11), who compared cloze completion probabilities with three different language models (ngram model, recurrent neural network and topic model) in predicting eye movements. They tested the hypothesis that each language model is more suitable for capturing a different cognitive process in reading, which in turn is reflected by early versus late eye-movement measures. Item-level analyses showed that the correlations of each eye movement measure were stronger with each language model than with cloze. In addition, fixation-event based analyses revealed that the ngram model better captured lag effects on early measures (replicating the results from Smith and Levy (10)), while the recurrent neural network more consistently yielded lag effects on late measure. A more recent study (22) found neural evidence for the advantage of language models over cloze, by showing that predictions from LLMs (GPT-3, ROBERTa and ALBERT) matched N400 amplitudes more closely than cloze-derived predictions. Such evidence has led to the belief that language models may be suitable for theory development in models of eye-movement control in reading (11).”

13. 6/2/2: Insert “our” after “advancing”.

We fixed it accordingly.

14. 8/2: I would remove the abbreviations for the eye-movement measures because they are not used elsewhere in the manuscript (except Figure 2, where they are provided). Also, I would list the measures with numbers; e.g., “… of interest were: (1) skipping; i.e., …” Do the same on p. 20.

Yes, you are right. Thank you for suggesting it. We have updated it accordingly.

*15. p. 12, Figure 3: The axes labels are too small to read. Also, the p-value associated with ** is not indicated in the note.*

Good to know the labels might be too small for some readers. We have updated the figure with a larger font for the axe’s labels. Also, thank you for noticing the missing information about the p-value. We have added it accordingly.

16. 14/3/4: “as-” --> “as”

We fixed it accordingly.

17. 14/3/7: Shouldn’t this read “semantic and syntactic processes” given the discussion on p. 6?

Yes, we agree. Thank you for pointing it out.

18. 15/2/5: Remove "specifically".

We removed it.

19. 15/2/6: "higher" --> "larger"

We fixed it accordingly.

20. 15/2/7-11: The argument beginning with "Given the model's assumption..." doesn't make sense to me. I would suggest clarifying this argument or removing it.

Thank you for pointing this out. We hope to have clarified the argument (in light of the systematic error analysis requested for revision) (Discussion, 20/2).

"One potential explanation involves the effect of word predictability on skipping being larger in OB1-reader than in human readers. The model skips highly predictable words more often than humans do. The high skipping rate seems to be related to the relatively slow first-pass reading observed in the simulations. The longer the model spends fixating a certain word, the more activity parafoveal words may receive, and thus the higher the chance that these are recognized while in the parafovea and are subsequently skipped. This effect can be more pronounced in highly predictable words, which receive more predictive activation under parafoveal preview. Thus, given the model's assumption that early (i.e. in parafoveal preview) word recognition largely drives word skipping, predicative activation may lead OB1-reader to recognize highly predictable words in the parafovea and skip them. Parafoveal recognition either does not occur as often in humans, or does not cause human readers to skip those words as reliably as occurs in the model."

21. 16/1/6: Shouldn't "model quality" be "model size"?

Model quality of LLMs in computational linguistics can refer to not only "model size" (which can actually refer to different settings, such as parameter count, number of layers, vocabulary size and training data size), but also to prediction accuracy (which can be operationalized in various ways, such as average word cross-entropy, model perplexity, and so on). We have clarified this point (22/2/9):

"Our results align with studies that have found language model word prediction accuracy, commonly operationalized as perplexity or cross-entropy, and model size, commonly operationalized as the number of parameters, to positively correlate with the model's psychometric predictive power."

22. 16/2/11: I think I understood what the authors meant by "failed falsification attempt" but I suspect that many readers won't. So please expand this point.

We have elaborated on this phrase (23/1/11):

"It at best is a failed falsification attempt, that is, we failed to show that language models are unsuitable for complementing cognitive models of reading. Our results rather suggest (…)"

23. 17/3: In the preceding paragraphs, the authors briefly describe how LLMs have been interpreted and then provide an argument for why each of these interpretations is limited. The authors don't do this for the interpretation mentioned in this paragraph, however. That said, is there any evidence supporting or refuting this interpretation?

Thank you for the excellent question. We have expanded on the interpretation mentioned by answering to your question (24/3/4):

"The capability of language models to encode linguistic knowledge has been taken as evidence that language - including grammar, morphology and semantics - may be acquired solely through exposure, without the need for, e.g., an in-built sense of grammar (42). How humans acquire language has been a continuous debate between two camps: the proponents of universal grammar argue for the need of an innate, rule-based, domain-specific language system (43,44), whereas the proponents of usage-based theories emphasize the role of domain-general cognition (e.g. statistical learning, (45), and generalization, (46)) in learning from language experience. Studying large language models can only enlighten this debate if those models are taken to capture the essence of human learning from linguistic input."

24. 18/1/2: Remove "and more" and "still".

We updated it accordingly.

25. 18/1/7: Again, I think I understand what the authors mean by "probing" the internal versus external behavior of neural networks, but I suspect that most readers won't. So briefly expand this point.

We agree with the reviewer that this point could be expanded for clarity (25/1/13):

"When certain input or architectural features can be associated with hypotheses about cognition, testing whether these features give rise to the observed model behaviour can help adjudicate among different mechanistic explanations."

26. 19/1/6: The claims that "cognitive models of reading often lack [accounts of semantics and discourse]" isn't quite accurate. I would suggest weakening this claim and citing a few of the discourse-processing models (e.g., Fletcher et al., 1996; Frank et al., 2007; Kintsch, 1998; Myers & O'Brien, 1998; van den Broek et al., 1996).

Thank you for pointing this out. Here, we were more specifically referring to models of eye-movement control in reading. We corrected the term to avoid confusion:

"Computational models of reading, and more specifically, eye-movement control in reading, are built (…)" (26/3/4)

"(…) which models of eye-movement control in reading often lack." (26/3/14)

27. 21/2/2: "7B" --> "7 billion"

We fixed it accordingly.

28. 21/2/4: "124M" --> "124 million"

We fixed it accordingly.

29. 23/1/1: Figure 1 is actually Figure 4.

We fixed it accordingly. Thank you for noticing it.

30. 25/2: In discussing the model's assumption about predictability, use numbers to list the assumptions; e.g., "…predictability being: (1) graded, …"

We updated it accordingly.

31. 26/3/4: Figure 2 is actually Figure 5.

We fixed it accordingly. Thank you for noticing it.

Reviewer #2

Pros:
- The paper introduces an novel integration of LLMs with a cognitive model, contributing to the field of reading research by providing a way of obtaining more dynamic and contextually aware word predictability scores.
- By outperforming traditional cloze methods, the study highlights the potential of LLMs to enhance predictive accuracy in reading simulations.
- The authors are very careful about interpreting the results, ensuring they do not fall into the trap of proposing LLMs as cognitively-plausible models.

Cons/questions:

- The authors only consider the first token of multi-token words, rather than considering the joint probability of all tokens that constitute the word. For example, "customary" is represented as two tokens in LLaMA's vocabulary, and the authors use only p(custom) as the predictability score for the word "customary" instead of p(custom)×p(ary|custom). I am not sure this is the best approach as it may overestimate the predictability of longer words. Furthermore, this creates discrepancy across different sources of predictability (e.g. with each new tokenizer, the representative part of a multi-token words will differ). The implication of this design choice must be discussed in the paper.

This is a great point. The reason we adopted a first-token-only strategy, in spite of the drawbacks mentioned by the reviewer, is that we do not only use the predictability of the

text words, but also of all the predicted words for each text position. We only know which tokens, and thus probabilities, constitute the text words, but not all the other potentially predicted words in each text position. The approach suggested by the reviewer would require us to keep feeding the language models with the predicted token and getting the next token until we seem to have formed a word. This should be done for every position in the text and every token predicted by each language model. Additionally, we would still run into the risk of thinking a word was predicted when a longer word was actually the predicted one, but we just stopped feeding the language model before we could see another word was "intended". To avoid running into these issues, we simplified the approach using the first-token-only strategy. Nevertheless, we agree with the reviewer that the implications of this design choice should be discussed. We incorporated the great points brought up by the reviewer to the Language Models subsection of the Methods (page 31):

"We are aware that this design choice may overestimate the predictability of long words, as well as create discrepancies between different sources of predictability (as different language models have different tokenizers). However, in the proposed predictability mechanism, not only the text words are considered, but also all the words predicted at a given text position (above a pre-defined threshold). Other approaches that aggregate the predictability estimates over all tokens belonging to the word, instead of only the first token, would require computing next-token predictions repeatedly for each different predicted token for each text position, until we assume a word has been formed. To avoid these issues and since the proportion of text words which are split into more than one token by the language models is moderate (17% and 16% from LLaMA and GPT2, respectively), we adopted the simpler first-token-only strategy."

- Although I do not have a problem with it, the choice of GPT-2 and LLaMA should be justified. GPT-2 is not traditionally regarded as an LLM (it is perhaps the last 'small' language model), so it could be presented as a baseline language model to demonstrate the potential benefits of more complex LLMs. It would also be interesting to see whether models with increasing sizes yield better performance or if there is a potential limit to the benefits of LLMs at a certain parameter count.

We agree with the reviewer that the choice of language models should be justified and thank them for the great suggestion. Indeed, the idea was to compare a simpler transformer-based language model with a more complex transformer-based language model. GPT-2 is part of the transformer-based generation of language models trained on a relatively large amount of data (8M web pages) and is often contrasted to BERT, whose base version has also around 100M parameters. Even though they are obviously smaller than the current state-of-the-art language models, as far as we know, they are still considered large language models (e.g., even GPT-1 is on wikipedia's LLM list), especially when compared to older types of language model architectures, such as recurrent neural nets and ngrams. We now explain the decision of using GPT-2 and LLaMA more explicitly (pages 10-11):

"The LLM values were generated from GPT2 and LLaMA. We compare the performance of a simpler transformer-based language model, i.e. GPT-2, with a more complex one, i.e. LLaMA. Both models are transformer-based, auto-regressive LLMs, which differ in size and next-word prediction accuracy, among other aspects. The version of GPT2 used has 124 million

parameters and 50k vocabulary size. The version of LLaMA used has a much higher number of parameters, 7 billion, but a smaller vocabulary size, 32k. Importantly, LLaMA yields a higher next-word prediction accuracy on the Provo passages, 76% against 64% by GPT-2 (see Language Models in Methods for more details on these models)."

Furthermore, we find the suggestion of experimenting with parameter counts an interesting one. One of the differences between GPT-2 and LLaMA is in the number of parameters. But there are also other differences, which weakens the claim that there is a relation between model size and quality of predictability estimates for reading simulations. Indeed, a better set up would be to hold all other model variables constant and only change the parameter count. We mention this idea for future research with a focus on finding the ideal language model settings, including parameter count, for providing predictability estimates for reading simulations (page 22):

"Note that number of parameters and next-word prediction accuracy are not the only differences between the models used. Further investigation is needed comparing more language models, and the same language model with one setting under scrutiny which varies systematically (e.g. all settings are the same except the parameter count), to help to determine which language model and settings are best for estimating predictability in reading simulations. Our results suggest that more complex pre-trained LLMs are more useful to this end."

- The methodology section could benefit from more details. The threshold of 0.01 feels rather arbitrary (how many continuations did you get for 0.005 or 0.001?). It is generally advisable to re-normalize the probabilities after any kind of filtering to ensure they still sum to one. Did you apply softmax again to the probabilities of the remaining words? Having normalized true probability distributions is important for accuracy, especially when comparing different sources.

We thank the reviewer for pointing this out. The threshold is indeed rather arbitrary. Nonetheless, we did follow some criteria to define the threshold, as described on page 21 of the unrevised manuscript. We now have added a more detailed description of the rationale in response to your very good questions (pages 29-30 of revised manuscript):

"We have attempted a few threshold values (low = 0.001, medium-low = 0.005, medium = 0.01, medium-high = 0.05 and high = 0.1). The medium threshold (0.01) provided the closest number of continuations and average top-1 predictability estimate to those of cloze. For example, using the medium threshold on GPT-2 predictions provided an average of approximately 10 continuations (range 0-36) and an average predictability of 0.29 for the top-1 prediction, which was the closest to the values from cloze (average of 15 continuations ranging from 0 to 38, and top-1 average predictability of 0.32). Low and medium-low provided a much higher number of continuations (averages of 75 and 19, ranging up to 201 and 61, respectively), whereas medium-high and high provided too few continuations compared to cloze (average of 2 and 1 continuations, ranging up to 12 and 5, respectively). The medium threshold was also optimal for LLaMA. Note that we have not applied softmax to the resulting sequence of predictability estimates. The highly long tail of predictability

estimates excluded (approximately the size of the LLM vocabulary, i.e. ~50k estimates for GPT-2 and ~32k for LLaMA) meant that re-normalizing the top 10 to 15 estimates (the average number of continuations post threshold filtering) would remove most of the variation among the top estimates. For instance, in the first passage the second word position led to likelihoods for the top 12 predictions varying between .056 and .011. Applying softmax resulted in all estimates transformed to .08 when rounded to two decimals. Thus, even though it is common practice to re-normalize after filtering to ensure the values sum to one across different sources, we opted to use predictability estimates without post-filtering re-normalization."

Overall, this paper makes a contribution to the field by showing how the LLMs can be integrated into the existing cognitive modeling techniques. The use of LLMs to predict word predictability in reading contexts perhaps will lead to more research in reading comprehension.

Thank you for your comments on our work. We are glad to read you find it novel and are positive about our caution in interpreting the results. We revised the manuscript according to your thoughtful questions and suggestions and believe the changes have improved it.

Reviewer #3

1. I'm curious whether it's possible to vary the size of the context window of the LLMs as an experimental parameter to see how larger/smaller contexts influence the gaze metrics, specifically in relation to early vs late measures.

This could in principle be an interesting venue for investigation into whether a transformer-based LLM could account for different cognitive processes (early vs. late) during reading. The idea of LLMs processing the text here and giving predictability estimates is that the input should be as close as possible to the input readers have while reading the text. Decreasing the size of the sequence/context given to the LLM would also mean that the predictability values output by the LLM wouldn't be based on the whole text read so far, which we assume is the case for the readers. Hofmann et al. (2022) have done something similar to the reviewer's suggestion, but using different types of language model architecture that can process different sizes of the context. They did not include transformer-based language models. Such an experiment would definitely require more careful thinking to determine what changing the context size in a transformer-based LLM in which each time step, or sequence given, is independent of each other, could tell us about the cognitive processes underlying reading. We mention the reviewer's suggestion as a potential venue for future work (page 26, paragraph 2).

2. Page 6. The sentence starting with: "This evidence has led to believe..." seems to be missing a subject (but I'm not a native English speaker so I might be wrong, but it sounds a bit strange to me).

We have corrected the sentence:

"This evidence has led to **the belief** that LLMs may be suitable (…)".

3. I understand it is out of scope for this article but it would be interesting to compare results also across different types of models of eye movements during reading (e.g., SWIFT and EZ Reader). This way one might get a clearer understanding whether LLMs are generally better than Cloze estimates independently of the particular mechanisms of the proposed eye movement model, or alternatively, if the results might depend more on the mechanistic details of the particular model.

Interestingly, this comment connects well with a point we raised in the penultimate paragraph of the Discussion, in which we put forward our belief that the success of LLMs here is likely dependent on our proposed mechanism. We do believe your suggestion is a good point for future work, so we now mention this as a venue for future work in the same paragraph (page 25/paragraph 2):

"More investigation is needed as to what extent the benefit of LLMs is independent of the particular model of reading or mechanism proposed. Analyzing the effect of LLM-derived predictability in other models of reading, especially serial ones (e.g. E-Z Reader) could provide a clearer understanding of the generalizability of such approach."

4. Page 9. "These results suggest that language models, especially with higher prediction accuracy, are good word predictability..." So there is a clear relationship between an LLMs general prediction accuracy and the fit to eye movement data? Maybe some simple comparison metrics between GPT2 and LLaMa would help the reader along the way here. For the reader it is not clear at this point in the text how these two LLMs differ, for example in terms of vocabulary size or overall prediction accuracy.

We agree with the reviewer that it is not clear at this point in the text how the two models differ, as this is only explained in Methods and Supporting Information. We thank the reviewer for the suggestion on adding comparison metrics between the LLMs. We have added information on the models to the Results which we believe are sufficient to help the reader understand the claims made in this section (pages 10-11):

"The LLM values were generated from GPT2 and LLaMA. We compare the performance of a simpler transformer-based language model, i.e. GPT-2, with a more complex one, i.e. LLaMA. Both models are transformer-based, auto-regressive LLMs, which differ in size and next-word prediction accuracy, among other aspects. The version of GPT2 used has 124 million parameters and 50k vocabulary size. The version of LLaMA used has a much higher number of parameters, 7 billion, but a smaller vocabulary size, 32k. Importantly, LLaMA yields a higher next-word prediction accuracy on the Provo passages, 76% against 64% by GPT-2 (see Language Models in Methods for more details on these models)."

5. Page 12, fig 3. Please specify which statistical hypothesis test twas used to derive the p-values.

We thank the reviewer for spotting the missing information. We have added which statistical test was used (page 11/paragraph 3):

“To check whether the simulated eye movements across predictability estimators were significantly different ($p <= .05$), we ran the Wilcoxon T-test from the Scipy python library.”

6. Page 14, "the current paper is the first to evidence..." Sounds grammatically strange to my ears.

We agree with the reviewer and have updated the sentence:

“The current paper is the first to **reveal** that large language models (LLMs) can complement cognitive models of reading at the functional level.”
